# A High-Throughput Method for the Comprehensive Analysis of Terpenes and Terpenoids in Medicinal Cannabis Biomass

**DOI:** 10.3390/metabo10070276

**Published:** 2020-07-06

**Authors:** Christian Krill, Simone Rochfort, German Spangenberg

**Affiliations:** Agriculture Victoria, AgriBio, Centre for AgriBioscience, Bundoora, VIC 3083, Australia; simone.rochfort@agriculture.vic.gov.au (S.R.); german.spangenberg@agriculture.vic.gov.au (G.S.)

**Keywords:** cannabis volatiles, terpenes and terpenoids, profiling, high-throughput method

## Abstract

Cannabis and its secondary metabolite content have recently seen a surge in research interest. Cannabis terpenes and terpenoids in particular are increasingly the focus of research efforts due to the possibility of their contribution to the overall therapeutic effect of medicinal cannabis. Current methodology to quantify terpenes in cannabis biomass mostly relies on large quantities of biomass, long extraction protocols, and long GC gradient times, often exceeding 60 min. They are therefore not easily applicable in the high-throughput environment of a cannabis breeding program. The method presented here, however, is based on a simple hexane extract from 40 mg of biomass, with 50 μg/mL dodecane as internal standard, and a gradient of less than 30 min. The method can detect 48 individual terpenes and terpenoids and was validated for selectivity, linearity, LOD/LOQ, precision, intermediate precision, and accuracy (recovery) for 22 terpenes and terpenoids. The validation parameters are comparable to previously published studies that employ significantly longer runtimes and/or more complex extraction protocols. It is currently being applied to medicinal cannabis precision breeding programs.

## 1. Introduction

*Cannabis* is a genus of annual dioecious plants within the family Cannabaceae with a rich and complex constituency of pharmacologically relevant phytochemicals [1,2]. Under the Single Convention on Narcotic Drugs (1961), cannabis was deemed to be a plant without medicinal purpose, a conclusion based on very little scientific evidence or clinical trial data [3]. As a result, medicinal use of cannabis was practically prohibited by its status as an illegal narcotic worldwide, and research into cannabis chemistry and biology was largely limited to law enforcement and forensics applications [4]. Changes in the attitudes towards cannabis across various societies and increasing evidence for the benefits of cannabinoids in the treatment of otherwise intractable conditions have precipitated recent changes to legislature in a number of jurisdictions, including Australia [3]. Interest in the medicinal uses of cannabis is increasing, as indicated by a growing number of review articles on the topic [5,6,7,8,9].

Cannabis, cannabis extracts, and purified (to varying degrees) major cannabinoids Δ9-tetrahydro cannabinol (THC) and cannabidiol (CBD) are now being used for the treatment of intractable and debilitating illnesses, such as Dravet’s Syndrome, and investigated for a range of others [5,7,10,11,12,13]. When administered as medicinal cannabis extracts, that is, along with the remaining portion of other cannabinoids and non-cannabinoid phytochemicals, less than a quarter of the equivalent dose of purified CBD is required, and markedly lower side effects are commonly observed [7]. This is in line with a long line of anecdotal evidence that complex plant-based medicines are more effective than their isolated “actives”, and has given rise to the concept of the “entourage effect”—the modulating or synergistic effects of phytochemical compounds, such as the minor cannabinoids on the effect of THC [14,15,16]. Despite a current lack of understanding for the underlying mechanisms [17], the concept is increasingly applied to non-cannabinoid cannabis phytochemicals, such as terpenes and terpenoids [6,10,11,18].

Terpenes are a class of hydrocarbons synthesized from 5-carbon units, or “building blocks”—dimethylallyl pyrophosphate and isopentenyl pyrophosphate. Depending on the number of C5 units and possible substitutions, they are further classified based on number of units (e.g., C10 monoterpenes, two subunits, C15, sesquiterpenes, and three subunits) or functional groups (terpenoids and oxygenated). Mono- and sesquiterpenes are classified as volatile and semi-volatile compounds, respectively, and higher order terpenes (e.g., C20 diterpenes and C30 triterpenes) exist as steroids, waxes, and resins. Cannabis mono- and sesquiterpenes are responsible for the characteristic smell of the plant and its products. They are also present at pharmacologically relevant concentrations [16,18,19,20], and a growing body of evidence suggests they have pharmacological properties in their own rights [21,22]. Their common use as food additives and in cosmetics products highlights the inherent safety of these compounds [21].

As Leghissa and colleagues note [23], conclusively identifying terpenes in cannabis is challenging due to the large variety of possible candidates and a lack of commercial standards for a large number of them. With the increased interest in medicinal cannabis and the contribution of terpenes to the entourage effect, methods to quantify terpenes in medicinal cannabis biomass and products are becoming more available in the literature [24,25,26,27]. Often, these methods require large quantities of biomass (1–5 g), use large quantities of organic solvents (up to 100 mL/sample), and include separations with gradient runtimes exceeding 60 min. These methods are therefore not easily applied in a high-throughput manner. To facilitate terpene quantitation and profiling in order to enable accelerated precision breeding programs in medicinal cannabis, we developed a fast (30-min separation), microscale (40 mg sample), high-throughput gas chromatography–mass spectrometry (GCMS) terpene profiling and quantitation method.

## 2. Results

### 2.1. Sampling Techniques

**Figure 1:**
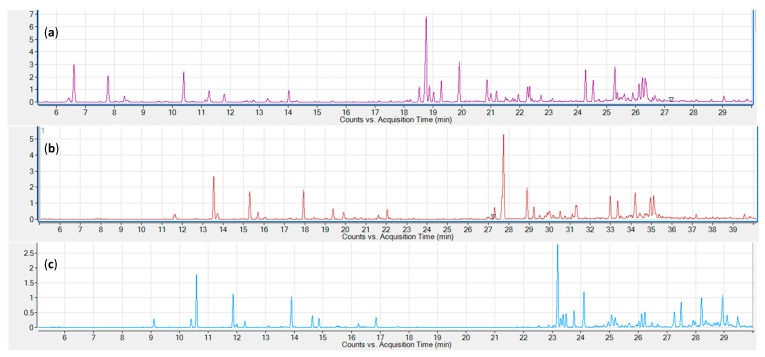
Comparison of three cannabis volatile sampling techniques. (**a**) static headspace; (**b**) solid-phase microextraction (SPME); (**c**) liquid extract (Hexane).

Three common sampling and sample introduction techniques were evaluated—static headspace, solid-phase microextraction (SPME) and liquid injection of an organic solvent extract. Representative chromatograms for the three tested sampling techniques are shown in Figure 1. All three techniques provide excellent signal strengths for the lower boiling monoterpenes. Sesquiterpenes are underrepresented in the static headspace chromatogram (a), while the SPME chromatogram shows a stronger signal for early eluting sesquiterpenes. Higher boiling sesquiterpenes are only adequately represented in the hexane extract chromatogram.

### 2.2. Columns

**Figure 2:**
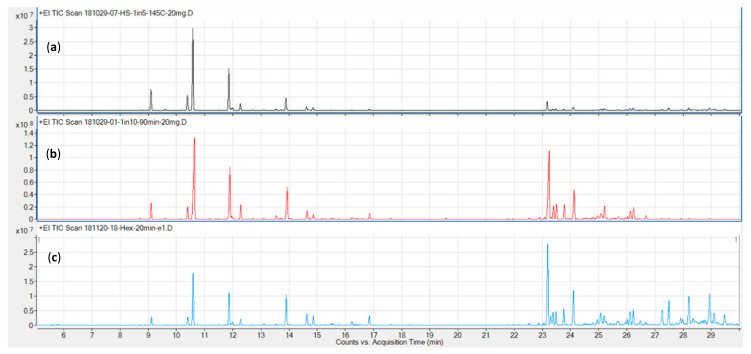
Representative chromatograms for the three columns tested: (**a**) DB17; (**b**) VF-35; (**c**) DB-5.

Representative chromatograms for the three columns tested (DB5, DB17, and VF35, in order of increasing polarity) are shown in Figure 2. The DB5 (panel c) and DB17 (panel a) columns performed comparably well in terms of resolution and overall run time (≤ 30 min), while the VF 35 column required a runtime in excess of 35 min for the chosen temperature program.

### 2.3. Solvent Optimisation

**Figure 3:**
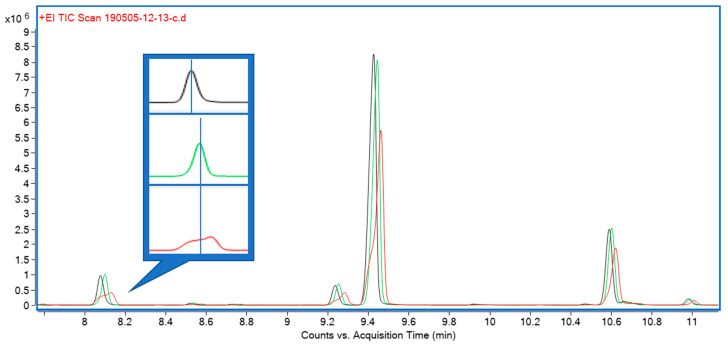
Comparison of peak shapes for early eluting compounds using three different injection solvents: hexane (black), ethyl acetate (green), isopropanol (red). Callout: enlarged detail of peaks at 8.05 min, separated, with vertical line through apex highlighting symmetry; solvents other than hexane cause undesirable peak shapes.

Three solvents—hexane, isopropanol and ethyl acetate—were initially trialed for liquid extraction. All three performed equally well in terms of extraction efficiency, but both isopropanol and, to a lesser extent, ethyl acetate were causing early eluting compounds to show undesirable peak shapes during GC analysis. A comparison of peak shapes is shown in Figure 3. Furthermore, hexane extracts appeared as light green, clear extracts, whereas ethyl acetate and isopropanol extracts were opaque and of a much darker color, indicating the presence of a higher proportion of non-target compounds.

### 2.4. Compound Identification and Resolution

A total of 48 individual compounds were detected across multiple strains used. Of those, 22 were monoterpenes and -terpenoids, with the remainder consisting of sesquiterpenes and terpenoids. Compound names, base peak (quantifier) ion *m*/*z*, retention times and indices, and identification status are summarized in Table 1 Most monoterpene identities predicted by spectral library matching were confirmed using authentic standards, with the exception of fenchol and trans-2-pinanol. The α thujene, β-phellandrene and thujanol identities were confirmed by matching published retention times indices (RIs). Sesquiterpene preliminary identities were confirmed using authentic standards for 8 out of 25 compounds (Table 1), and RI matching for a further 3.

Resolution was calculated based on Equation (1). Values presented in Table 1 are based on the respective base peak (qualifier) ion and are given for the closest preceding (R_p_) and following peak (R_s_).

### 2.5. Linearity

A combined cannabis terpenes (CT) and sabinene standard was injected at ten concentrations from 100 μg/mL to 0.195 ng/mL, based on a 1:2 dilution series of the 100 μg/mL standard. All calibration curves obtained were linear with R^2^ values greater than or equal to 0.994. Based on individual detection limits, the curves spanned all ten calibration points, except linalool, ocimene and trans-caryophyllene (100–0.391 μg/mL), caryophyllene oxide, guaiol and isopulegol, (100–1.56 μg/mL) and α-bisabolol and β-*cis*-caryophyllene (100–12.5 μg/mL). Nerolidol was removed from the analysis due to poor peak shapes and high detection limits.

### 2.6. Detection and Quantitation Limits

LOD and LOQ were determined as LOD = 3.3 × σ/S and LOQ = 10 × σ/S, with σ representing the standard deviation of the compound and S the slope of the compound’s calibration curve. Detection limits range from 0.017 μg/mL to 1.144 μg/mL (Table 2); quantitation limits range from 0.052 to 3.468 μg/mL (β-pinene and α-bisabolol for both LOD/LOQ)

### 2.7. Accuracy

To determine method accuracy, three replicates of a sample preparation were spiked with 0.5, 0.25, and 0.05 mg/g of CT and sabinene and extracted as described above. Unspiked biomass was extracted to determine endogenous levels. The recovery requirements of the spike as expressed in % adjusted for endogenous levels were 80–120%. Measured values ranged from 61.8% to 121.4%, with the majority of compounds ranging from 80% to 120% recovery across the three concentrations (Table 3).

### 2.8. Precision and Intermediate Precision

Eight individual preparations of biomass were extracted independently by two analysts on different days and analyzed separately. Relative standard variations (%RSD) varied from 1.13 to 8.74 for single analyses, 1.65 to 7.54 for both analyses combined (Table 4). Three compounds—α-terpinene, eucalyptol, and terpinolene—were found to have %RSDs > 10. Results are summarized in Table 4. Myrcene content was determined from a 1:5 dilution of the original extract to bring the concentration back into the linear range.

## 3. Discussion

In recent years, the push from society to employ medicinal cannabis in the treatment of intractable and debilitating illnesses has been met by some governments legalizing research into medicinal cannabis [3,4,5]. Research in this area has escalated but the understanding of the complex composition of cannabis extract is still rudimentary [17]. In one such program, we seek to profile and measure terpenes and terpenoids in medicinal cannabis inflorescence through GCMS to inform genome-wide association studies (GWAS) and other aspects of medicinal cannabis accelerated precision breeding for the development of novel designer strains. The method should be:

- Comprehensive—detecting the highest possible number of individual compounds.
- Quantitative—reliably providing absolute quantitation for as many compounds as possible.
- High-throughput—processing the maximum number of samples possible in as short a time as possible, from as little biomass as possible.

For our purposes, absolute quantitation, while desirable, is not strictly a prerequisite, as meaningful correlations to genomics data can be drawn from relative quantities, i.e., analyte-internal standard response ratios. Similarly, baseline separation, defined as R > 1.5 and generally a requirement for validated methods, is not our priority. As we are expecting close to 1000 offspring from 2–3 individual crossing events, resulting in 5000 potential samples (including replication), increasing sample throughput over existing methods was a major consideration.

We evaluated a range of sample extraction and introduction strategies, two headspace techniques (static headspace and solid-phase microextraction (SPME)) and solvent extraction. SPME was evaluated for its ease of use and comparably high sensitivity [28]. It has been used extensively for volatile profiling in cannabis and cannabis products [29,30], and has been reviewed [23]; however, to our knowledge, none of these methods were validated for quantitation. Most of our preliminary identification work was carried out using SPME, and the method reliably provided very strong signals for all monoterpenes and earlier eluting sesquiterpenes. Higher boiling sesquiterpenes were present, but their signals were comparatively weaker, most likely due to insufficient volatility at the extraction temperature or insufficient fiber incubation. Higher extraction temperatures (> 100 °C) may aid in volatilizing these higher boiling compounds, but will most likely result in a loss of more volatile monoterpenes from the fiber and longer exposure times (60–120 min, e.g., [31]) would reduce sample throughput.

Higher extraction temperatures (145 °C) were employed for static headspace (SHS) analysis. This methodology is generally regarded as providing superior quantitation compared to SPME and has been described previously for the quantification of terpenes in cannabis [32,33]. We found that, for our purposes, it lacked sensitivity for all sesquiterpenes, despite the higher extraction temperature.

The third sample extraction and introduction trialed was liquid extraction with an organic solvent. Solvents tested were hexane, ethyl acetate, and isopropanol. While all performed equally well in terms of their extraction characteristics, isopropanol and, to a lesser extent, ethyl acetate caused peak fronting, particularly for early eluting monoterpenes (Figure 3). Hexane also afforded clear, lighter colored extracts which, in our experience, translates to lower amounts of non-target compounds and nonvolatile contaminants, resulting in higher instrument uptime and reliability. Hexane was therefore chosen for subsequent experiments. Comparing chromatograms for the three extraction strategies, it became apparent that only solvent extraction was able to provide a full representation of all relevant mono- and sesquiterpenes and -terpenoids (Figure 1). In terms of throughput, headspace techniques would be advantageous, as they require minimal sample preparation (weighing); however, the hexane extract strategy still allows for well over 100 samples to be processed within a work day, equivalent to several days’ worth of instrument time. It was therefore decided to use solvent extraction with hexane for subsequent experiments.

Next, two additional columns were evaluated for their chromatographic characteristics. The initial method development described above was carried out on a DB-5 non-polar column. Other columns evaluated from our inventory were DB-17 and VF-35, both of intermediate polarity. A 5 °C/min gradient was chosen as a trade-off between shortest possible run time and best possible resolution. The runtime achieved is shorter than that reported by Giese and colleagues using a 3.5 °C gradient [24], while also resolving five additional, later eluting sesquiterpenes, including the validation compounds guaiol and bisabolol. Furthermore, by employing column backflushing after elution of bisabolol, we can prevent cannabinoids, column bleed, and other high-boiling compounds from entering the MS, reducing contamination of the source and therefore the need for more frequent source cleaning. Using a DB-17 column may also marginally reduce the runtime, as demonstrated in Figure 2 but would mean a trade off in terms of compound identification, as this column type is very rarely used to calculate retention times indices. Initial tests also showed it did not offer significant improvements in terms of resolution over the DB-5, which affords baseline or near-baseline separation for the validation compounds (Table 1). Notably, the resolution values here were calculated using extracted ion chromatograms (EICs) from a combination of standard and sample runs, which we believe is a more accurate representation of actual cannabis samples than presenting resolution data on standard runs alone [25,34]. The *p*-cymene, limonene, and eucalyptol eluted in close proximity in the total ion chromatogram (TIC), but are nonetheless resolved due to their unique quantifier ions. Furthermore, only limonene was detected in significant quantities in the available biomass. Based on the samples used for determining precision, the amount of eucalyptol and β-phellandrene was approximately 1.5% that of limonene each, while that of *p*-cymene was below the detection limit (cp Table 4).

A total of 49 distinct individual compounds were detected using this method. Of these, 23 were classified as monoterpenes and -terpenoids, the remainder as sesquiterpenes and -terpenoids. Compounds were assigned putative identities based on spectral library matching and retention time indexing. As far as possible, these predictions were confirmed using authentic standards. While monoterpene standards are readily available commercially, sesquiterpene standards have proven more difficult to obtain, an issue also encountered in other studies [6,32,35]. With the standards available to us, we were able to conclusively identify 18 out of the 23 monoterpenes, and 8 out of 26 sesquiterpenes, as summarized in Table 1; for a further 2 monoterpenes (β-phellandrene and 4thujanol) and 3 sesquiterpenes (bergamotene isomer 3, (−)-α selinene and δ-guaiene), we found matching retention time indices in the NIST webbook. The α-thujene was initially identified as α-phellandrene, based on spectral library matching, but did not match the retention time of an authentic α-phellandrene standard, and was assigned its current identity based on the RI published by Ascrizzi and colleagues [29].

The method was validated for 22 compounds, as listed in Table 5, for specificity (resolution) linearity, quantitation and detection limits, accuracy (recovery), and precision. Nerolidol was removed from the validation early due to poor sensitivity. Detection and quantitation limits for the remaining 21 compounds ranged from 0.017 (LOD) and 0.052 μg/mL (LOQ) for β-pinene to 1.144 and 3.468 μg/mL for bisabolol. The majority of compounds can be detected at less than 0.1 μg/mL in solution, equivalent to 2 ppm in biomass (*w*/*w* dry weight), and quantitated at less than 0.2 μg/mL, equivalent to 4 ppm in biomass. All LODs/LOQs were well below what is generally considered pharmacologically relevant (500 ppm) [18], and the responses were linear for the ranges tested. Using an MS/MS approach, as advocated by Shapira and colleagues [32] instead of acquiring scan data, was considered initially and may provide lower detection limits but also prevent the re-examination of data for previously “unexpected” compounds.

Accuracy was determined by spiking cannabis samples with three concentrations of the validation terpenes within the linear range and adjusting for endogenous levels. Recoveries mostly fall in the acceptable 80–120% range, with the exception of isopulegol (61.8% high; 66.2% mid), β*cis* caryophyllene (62.0% high; 72.9% mid), and caryophyllene oxide (69% high; 76.3% mid). Interestingly, the values for the low spike concentration for these compounds do fall within the 80–120% range. The precision of the method as determined by the %RSD for eight sample preparations, was generally between 1% and 10%, with the exception of α-terpinene (16.81% Analyst 1; 7.86% Analyst 2) and eucalyptol (20.86% Analyst 1; 13.55% Analyst 2). Four validation compounds—3-carene, *p*-cymene, ocimene, and nerolidol—were below detection limits entirely in the cannabis material and therefore not part of the precision determination.

Given the large concentration range of terpenes in cannabis, we consider these values acceptable for our purpose. The validation parameters obtained with this method are comparable to previously published studies that use much longer gradient times and that are therefore less suitable for high-throughput terpene profiling [25,32]. As Shapira and colleagues note [32], the actual accuracy and precision can be influenced by minor variations in sample preparation and variation in endogenous levels. Consequently, we saw the largest outlier in precision in two of the lowest level compounds.

## 4. Materials and Methods

All solvents were 99% purity or better and obtained from Thermo Fisher Scientific (Scoresby, VIC, Australia). Supelco 30/50 DVB/CAR-PDMS SPME fibers were purchased from Sigma-Aldrich (Ryde, NSW, Australia).

Unless stated otherwise, individual authentic standards and standard solutions for compound identification and were obtained from Sigma-Aldrich and Leco Australia (Baulkham Hills, NSW, Australia) as distributers for Restek (Bellefonte, CA, USA). Combined cannabis terpenes standard solutions #1 and #2 (CT, 2.5 mg/mL in isopropanol) were purchased from Restek. Dodecane (internal standard) and C_8_-C_20_ alkane mix were obtained from Sigma-Aldrich.

Cannabis plants were grown at an Agriculture Victoria Research indoor growth facility. Multiple cannabis strains from a variety of chemotypes were used for the study. Dried samples of inflorescence were ground to a fine powder using a SPEX SamplePrep 2010 Geno/Grinder for 1 min at 1500 Hz.

In a preliminary experiment, different cannabis strains were analyzed for their volatile production and compared using principal component analysis. Chemically diverse strains were selected and combined to create a test sample that represents the largest possible variety of volatile compounds.

### 4.1. Headspace Techniques

The 20 mg combined sample was weighed into a 20 mL headspace vial, sealed, and incubated at 40 °C for 10 min. For SPME analysis, a 1 cm 30/50 divinylbenzene-carboxen polydimethylsiloxane fiber (Supelco, Bellefonte, PA, USA) was incubated in the sample headspace for 30 min and desorbed in the GC inlet for 3 min. The fiber was reconditioned between samples.

For static headspace analysis, 1 mL of the gas phase was removed using a gas-tight headspace syringe and injected directly. Different incubation temperatures from 40–145 °C were trialed to maximize recovery of higher-boiling sesquiterpenes. The syringe was kept at 150 °C and flushed with high-purity nitrogen between samples to avoid carryover.

### 4.2. Liquid Extracts

Of the combined sample, 40 mg was weighed into 2 mL microcentrifuge tubes, and 400 μL of extraction solvent (50 mg/L dodecane in hexane) were added. Samples were vortexed for 30 s, then sonicated for 10 min, and centrifuged at maximum *g* for 5 min. The supernatant was removed to an autosampler vial, the extraction procedure was repeated, and first and second extracts combined. A third extraction was used to determine extraction efficiency. Hexane, isopropanol, and ethyl acetate were evaluated as extraction solvents, and ultimately, hexane was chosen for the validation.

For measuring myrcene concentration, samples were further diluted 1:5 in hexane to bring the analyte concentration into the linear range.

### 4.3. Standard Preparation

For compound identification, individual standards were diluted in hexane (Sigma standards) or methanol (Restek individual standards) to between 20–100 ng/μL. The combined CT standards were used for quantitation, spiking and LOD/LOQ experiments. Dodecane and sabinene stock solutions were prepared at 10 mg/mL. CT standards #1 and #2 and sabinene stock solution were combined in hexane to obtain a solution of 100 μg/mL. This solution was then serially diluted 1:2 in hexane nine more times to obtain a lowest concentration of 0.195 μg/mL. To these, internal standard was added to a final concentration of 50 μg/mL.

### 4.4. GC–MS Analysis

All analyses were performed on an Agilent 7890A GC with a 7000 Triple Quadrupole Mass Spectrometer and Gerstel MPS autosampler with heated agitator. The GC inlet was held at 270 °C, with a split ratio of 20:1. The injection volume was 2 μL. Column 1 was an Agilent J&W DB-5MS-DG, 30 m × 0.25 mm I.D, × 0.25 μm film thickness with an integral 10 m guard column, connected to a 2.5 m × 0.18 mm deactivated fused silica capillary (noncoated) via a purged union. Other primary columns tested were Agilent J&W DB-17 30 m × 0.25 mm, × 0.25 μm, and Varian VF-35 30 m × 0.25 mm, ×1.0 μm. Helium was the carrier gas at a constant flow of 32 cm/s for column 1 and constant pressure of 8 psi for column 2. A single linear gradient was used starting at 60 °C held for 2 min and increasing to 200 °C at 5 °C /min. During post run time column 1 was backflushed for 5 min (approximately 5 column volumes) at 320 °C.

The MS temperatures were as follows—transfer line and EI source: 280 °C; quadrupoles: 150 °C. Data were acquired in MS1 scan mode from 40–250 *m*/*z* using EI at 70eV after a 5-min solvent delay. Compounds were putatively identified by spectral matching against the NIST 2008 library and retention time indices in the NIST webbook using the method of Van den Dool and Kratz [36]. Predictions were confirmed using authentic standards where available. Quantitation was performed using Agilent MassHunter Quant software with the largest fragment ion (base peak) used for quantitation.

### 4.5. Validation Parameters

The method was validated for resolution, linearity, quantitation and detection limits, accuracy, and precision for the compounds listed in Table 5.

Resolution (R) for the two adjacent peaks was determined using extracted ion chromatograms for the base peak ion (quantifier) of each detected compound, based on their retention times *T*_1_/*T*_2_ and peak widths *w*_1/_*w*_2_:

(1) $$R_{1,2}=2\frac{T_{2}-T_{1}}{w_{2}+w_{1}}$$

For determining linearity, LOD, and LOQ, a 1:2 dilution series of the combined CT and Sabinene standard, ranging from 100 μg/mL to 0.195 ng/mL, was injected. Calibration curves were required to have R2 values greater than or equal to 0.99. LOD and LOQ were determined as LOD = 3.3 × σ/S and LOQ = 10 × σ/S, with σ representing the standard deviation of the compound and S the slope of the compound’s calibration curve.

To determine method accuracy, three replicate sample preparations were spiked at 25, 12.5, and 2.5 μg/mL of CT and sabinene and extracted. Unspiked plant material was extracted to determine endogenous levels. Recoveries were determined as

(measured concentration − endogenous concentration)/spiked concentration × 100(2)

Acceptable recoveries were defined as 80–120%

Precision and intermediate precision were determined by analyzing eight individual preparations of plant material extracted independently by two analysts on different days. The relative standard deviation for the concentration of the compounds was ≤ 6% for individual analysts and ≤ 8% combined.

## 5. Conclusions

As accelerated precision breeding programs for medicinal cannabis become more widespread, accurately measuring the non-cannabinoid content in cannabis, particularly terpenes and terpenoids, has become increasingly important. Current methods, however, are unsuitable for the high-throughput analyses required for large scale breeding programs. The method presented here covers a large cross-section of commonly detected cannabis volatiles, is validated for a large proportion of compounds it covers, and offers significant improvement in terms of sample preparation and sample throughput over previously published studies.

## Figures and Tables

**Table 1:** Compounds detected in this study, their base peak (quantifier) ions (*m*/*z*), retention times (RT) and indices (RI), resolution to the closest preceding (R_p_) and following peaks (R_f_), and identification status (specID: based on library match; RI: based and library match and retention time index; confirmed: matches spectrum and RT of authentic standard; Val: confirmed compound used for method validation).

Name	*m/z*	RT [Min]	RI	R_p_	R_f_	Status
α Thujene	93.0	7.866	932	n/a	1.7	RI
α-Pinene (+/−)	93.0	8.056	940	1.9	1.6	Val
Camphene	93.0	8.505	958	4.8	1.7	Val
Sabinene	68.1	9.067	979	1.5	1.2	Val
β-Pinene (+/−)	69.2	9.215	985	1.3	1.4	Val
Myrcene	93.0	9.380	991	2.6	1.4	Val
3-Carene	79.0	10.007	1014	1.9	1.6	Val
α-Terpinene	119.0	10.230	1023	2.0	1.6	Val
*p*-Cymene	93.0	10.437	1031	4.1	1.9	Val
Limonene	121.0	10.565	1035	3.0	15.7	Val
β-Phellandrene	93.1	10.660	1039	1.2	1	specID, RI
Eucalyptol	93.0	10.678	1040	1.7	2.1	Val
Ocimene isomer	105.1	10.960	1050	1.8	2.2	Val
γ-Terpinene	93.1	11.367	1064	2.9	2.3	Val
4-Thujanol	93.1	11.750	1077	3.4	2.9	specID, RI
Terpinolene	93.0	12.150	1090	1.7	2.5	Val
Fenchone	81.1	12.333	1096	1.8	1.7	confirmed
Linalool	81.0	12.496	1101	1.4	2.7	Val
Fenchol	81.1	13.201	1128	7.4	2.0	specID
trans-2-Pinanol	93.1	13.421	1135	2.0	13	specID
Isopulegol	93.0	14.022	1156	1.6	2.8	Val
Borneol	95.1	14.758	1181	5.4	4.5	confirmed
Dodecane	57.1	15.370	1201	6.7	7.2	IS
Nerolidol	93.1	16.783	1253	2.6	22.5	Val
β-Bergamotene	119.1	21.015	1406	3.0	3.4	specID
α-Bergamotene	93.1	21.341	1420	3.6	2.3	specID
trans-Caryophyllene	93.1	21.600	1430	2.9	3.4	Val
g-Elemene iso1	121.0	21.768	1437	1.4	1.8	specID
Bergamotene iso3	119.0	21.848	1440	0.8	3.5	specID, RI
α-Guaiene	105.0	21.944	1444	1.7	1.8	specID
Farnesene	69.2	22.270	1456	4.1	2.1	confirmed
Humulene	69.1	22.524	1466	3.9	32.1	Val
epi-β-Selinene	93.1	23.396	1499	1.4	1.5	specID
(−)-α-Selinene	105.1	23.567	1506	1.7	1.1	specID, RI
Sesquiterpenes, coeluting	93.1	23.574	1506	1.5	1.4	specID
δ-Guaiene	161.0	23.662	1510	1.4	2.6	specID, RI
β-Guaiene	161.0	24.139	1530	1.2	1.5	specID
Farnesol	69.0	24.222	1534	3.2	3.7	confirmed
α-Bisabolene	93.1	24.512	1546	1.1	0.7	specID
Guaia-3,9-diene	161.1	24.572	1548	1.5	1.6	specID
3,7(11)-Selinadiene	161.1	24.680	1553	1.6	1.1	specID
β-*cis*-Caryophyllene	93.0	24.968	1564	2.5	4.1	Val
γ-Elemene iso2	121.1	25.152	1572	1.4	2.8	specID
Caryophyllene Oxide	93.0	25.691	1593	1.6	4.8	Val
Guaiol	93.0	25.970	1605	2.3	7.0	Val
β-Cadinene	189.1	26.666	1636	1.9	1.3	specID
γ-Gurjunene	59.1	27.400	1667	1.8	1.5	specID
Sesquiterpene	107.0	27.578	1675	1.2	1.4	specID
α-Bisabolol	93.0	27.989	1692	1.9	n/a	Val

**Table 2:** Detection (LOD) and quantitation (LOQ) limits in μg/mL for the validation compounds. Compounds are listed in order of their retention times. S: slope of calibration curve.

Compound	S	LOD	LOQ
α-Pinene (+/−)	0.039129	0.022	0.068
Camphene	0.023849	0.032	0.097
Sabinene	0.038856	0.026	0.079
β-Pinene (+/−)	0.047192	0.017	0.052
Myrcene	0.028807	0.047	0.142
3-Carene	0.030862	0.030	0.091
α-Terpinene	0.021459	0.038	0.115
*p*-Cymene	0.078714	0.021	0.065
Limonene	0.017349	0.066	0.200
Eucalyptol	0.008849	0.115	0.348
Ocimene	0.015782	0.057	0.174
γ-Terpinene	0.032899	0.046	0.139
Terpinolene	0.018898	0.045	0.137
Linalool	0.013307	0.060	0.181
Isopulegol	0.005376	0.045	0.136
trans-Caryophyllene	0.010502	0.092	0.326
Humulene	0.032275	0.021	0.065
β-*cis*-Caryophyllene	0.005202	0.388	1.177
Caryophyllene Oxide	0.004599	0.252	0.764
Guaiol	0.008546	0.107	0.324
α-Bisabolol	0.010087	1.144	3.468

**Table 3:** Recovery of analytes in spiked sample at three concentration levels.

	% Spike Recovery		
	High	Mid	Low
α-Pinene (+/−)	106.4	108.2	97.5
Camphene	112.6	121.4	119.0
Sabinene	108.8	115.7	98.7
β-Pinene (+/−)	110.7	113.7	102.2
Myrcene	106.5	104.8	95.5
3-Carene	112.4	118.7	115.7
α-terpinene	78.8	84.5	81.8
*p*-Cymene	112.2	115.6	116.5
Limonene	110.6	114.6	107.9
Eucalyptol	116.6	109.9	119.2
Ocimene	101	105.7	98.6
γ-Terpinene	104.6	108.9	101.6
Terpinolene	97.1	100.4	93.0
Linalool	113.6	114.5	105.5
Isopulegol	61.8	66.2	82.6
trans-Caryophyllene	93.5	99.1	89.7
Humulene	92.5	98.3	90.7
β-*cis*-Caryophyllene	62.0	72.9	87.7
Caryophyllene Oxide	69.0	76.3	84.8
Guaiol	102.6	104.1	93.3
α-Bisabolol	98.7	100.3	93.5

**Table 4:** Precision and intermediate precision results. Averages are expressed as μg/g dry biomass. Compounds not detected in the biomass used are marked as n/d.

	A1 Average	A1 %RSD	A2 Average	A2 %RSD	Combined Average	Combined %RSD
α-Pinene (+/−)	0.132	8.74	0.123	3.48	0.127	7.54
Camphene	0.035	8.27	0.033	3.61	0.034	7.01
Sabinene	0.005	5.07	0.005	2.54	0.005	3.92
β-Pinene (+/−)	0.233	7.56	0.221	2.95	0.227	6.17
Myrcene (1:5)	1.38	2.07	1.318	4.43	1.349	4.92
3-Carene	n/d					
α-Terpinene	0.005	16.81	0.005	7.86	0.005	13.86
*p*-Cymene	n/d					
Limonene	1.753	5.74	1.744	3.4	1.749	4.62
Eucalyptol	0.027	20.86	0.028	13.55	0.028	18.68
Ocimene	n/d					
γ-Terpinene	0.006	4.87	0.006	4.38	0.006	5.61
Terpinolene	0.015	10.26	0.014	3.73	0.014	7.23
Linalool	0.017	2.77	0.018	2.75	0.017	4.46
Isopulegol	0.06	2.43	0.06	1.97	0.06	2.52
Nerolidol	n/d					
*trans*-Caryophyllene	1.492	1.76	1.449	1.93	1.47	2.31
Humulene	0.419	2.44	0.408	1.66	0.413	2.6
β-*cis*-Caryophyllene	0.198	1.44	0.201	1.9	0.2	1.65
Caryophyllene oxide	0.121	2.79	0.129	2.11	0.125	3.8
Guaiol	0.479	1.86	0.491	2.13	0.485	2.57
α-Bisabolol	0.553	1.13	0.565	1.65	0.559	2.62

**Table 5:** Compounds used for method validation. Names, base peak ions (*m/z*), retention times (RT), and source are given. CT1: Restek cannabis terpenes standard mix #1 CT2: Restek cannabis terpenes standard mix #2, ind: individual standard (Sigma-Aldrich).

Name	*m*/*z*	RT	Source
α-Pinene (+/−)	93.0	8.056	CT1
Camphene	93.0	8.505	CT1
Sabinene	93.0	9.067	Ind.
β-Pinene (+/−)	93.0	9.215	CT1
Myrcene	93.0	9.380	CT1
3-Carene	93.0	10.007	CT1
α-terpinene	93.0	10.230	CT1
p-Cymene	119.0	10.437	CT1
Limonene	68.1	10.565	CT1
Eucalyptol	81.0	10.678	CT2
Ocimene isomer	93.1	10.960	CT1
γ-Terpinene	93.1	11.367	CT1
Terpinolene	93.1	12.150	CT1
Linalool	93.0	12.496	CT1
Isopulegol	121.0	14.022	CT1
Nerolidol	69.1	16.783	CT1
trans-Caryophyllene	93.0	21.600	CT1
Humulene	93.0	22.524	CT1
β-*cis*-Caryophyllene	69.2	24.968	CT1
Caryophyllene Oxide	79.0	25.691	CT2
Guaiol	105.1	25.970	CT1
α-Bisabolol	93.0	27.989	CT1
